# Association Between Trace Mineral Concentrations and Oxidative Stress in Children with ADHD Supplemented with Multinutrients

**DOI:** 10.1007/s12011-026-05017-5

**Published:** 2026-03-03

**Authors:** Lisa M. Robinette, Irene E. Hatsu, Chieh-Ming Wu, Olorunfemi Adetona, Alisha M. Bruton, Hayleigh K. Ast, James B. Odei, Brenda M. Y. Leung, Jeanette M. Johnstone, Ouliana Ziouzenkova

**Affiliations:** 1 Department of Human Sciences, The Ohio State University, 29 W. Woodruff Ave, 160 Ramseyer Hall, OH 43210 Columbus, USA; 2 Division of Environmental Health Sciences, College of Public Health, The Ohio State University, Columbus, OH USA; 3 Center for Mental Health Innovation, Psychiatry Department, Oregon Health & Science University, Portland, OR USA; 4 Division of Biostatistics, College of Public Health, The Ohio State University, Columbus, OH USA; 5 Faculty of Health Sciences, University of Lethbridge, Lethbridge, Canada Alberta; 6 OSU Extension, The Ohio State University, OH Columbus, USA

**Keywords:** ADHD, Oxidative stress, Antioxidant, Reactive oxygen species, Mineral, Multinutrient supplement

## Abstract

Oxidative stress (OS) arises from an imbalance between antioxidant defenses and the generation of reactive oxygen species (ROS), both of which are influenced by the levels and composition of trace elements. The Micronutrients for ADHD in Youth randomized controlled trial demonstrated behavioral improvements following 8 weeks of multinutrient supplementation in 54% of children compared to 18% on placebo. This secondary analysis examined the relationship between plasma minerals and OS-related parameters [biological antioxidant potential (BAP), glutathione peroxidase (GPx), glutathione reductase (GR), and reactive oxygen metabolites (ROM)] in 71 children (44 multinutrient, 27 placebo; median age 10.4 years). Correlation analysis revealed that selenium and zinc improved antioxidant response measured as GPx change in both treatment groups (*r* = 0.26, *p* = 0.03) and BAP in multinutrient group (*r* = 0.37, *p* = 0.01), respectively, while chromium change was inversely related to BAP (*r*=-0.41, *p* = 0.005) in the multinutrient group. BAP change was moderated by baseline selenium (β=-9.45, *p* = 0.006), zinc (β=-2.13, *p* < 0.001), and chromium (β = 68.9, *p* = 0.02), where children in the multinutrient group with low selenium, low zinc, and high chromium at baseline experienced BAP increases. Selenium also exhibited pro-oxidant effects: ROM change correlated positively with change in selenium in multinutrient group only (*r* = 0.34, *p* = 0.03). Baseline copper predicted ROM change (β = 0.213, *p* < 0.001), and ROM change correlated positively with changes in copper in both groups (*r* = 0.51, *p* < 0.001) and negatively with Mn (*r*=-0.24, *p* = 0.05), while iron did not moderate OS. Multinutrient supplementation may enhance antioxidant defenses in children who had low plasma zinc and selenium, while potential pro-oxidant effects in those with higher selenium and chromium warrant further investigation. **Clinical Trial Registry number for MADDY RCT**: NCT 03252522.

**Supplementary Information:**

The online version contains supplementary material available at 10.1007/s12011-026-05017-5.

## Introduction

Oxidative stress (OS) is increasingly implicated in neurodevelopmental disorders such as attention-deficit/hyperactivity disorder (ADHD) and autism spectrum disorder (ASD) [1–4]. Under OS, cellular antioxidant defences are insufficient to inactivate the reactive oxygen species (ROS) generated in the body, which can damage nucleic acids, lipids, and proteins [5]. Antioxidant systems defending against ROS include enzymes [e.g., superoxide dismutase (SOD), catalase (CAT), glutathione peroxidase (GPx), glutathione reductase (GR)], endogenous antioxidants [e.g., glutathione (GSH)], dietary antioxidant vitamins [vitamin E, vitamin C, etc.], and other bioactive antioxidants. OS can be quantitatively assessed by measuring overall pro-oxidant levels [derivatives of reactive oxygen metabolites (ROM)] and non-enzymatic antioxidant capacity [biological antioxidant potential (BAP)] [6]. In children with ASD, with and without co-occurring ADHD, increased ROM are associated with diagnosis and symptom severity of ASD [7–9]. Accumulating evidence indicates that in children with ADHD, elevated levels of OS are observed when compared to children without ADHD [1, 10, 11], suggesting that OS is a potential target for prevention or treatment of ADHD.

Trace minerals serve as cofactors in antioxidant enzymes [e.g., selenium in GPx; copper, zinc, and manganese in SOD; and iron in CAT] [12, 13], while chromium impairs SOD activity [14]. Additionally, copper and iron are principal catalysts for auto-oxidation and are enzyme co-factors involved in the synthesis and degradation of neurotransmitters [15]. Notably, the excess of iron, copper, or other metals such as chromium, manganese, nickel, and cobalt above physiological requirements lead to free radical formation via Fenton or Fenton-like reactions resulting in the depletion of antioxidants and contributing to OS [16]. Trace mineral levels, most commonly Zn, Fe, Cu, Mn, and Mg, have been broadly studied in ADHD [17–22]. Consistent evidence has shown that children with ADHD have lower serum ferritin, an iron-binding storage protein, compared to typically developing children, indicating altered iron metabolism [21, 23]. Despite evidence of abnormalities in trace mineral levels and OS in ADHD, the impact of these minerals on oxidative and antioxidant mechanisms has not been examined in children with ADHD.

Three previous clinical trials: one in adults, two in children, have studied multi-vitamin/mineral supplementation (“multinutrient”) to treat symptoms of ADHD and emotional dysregulation [24–26]. Results from an 8-week double-blind placebo-controlled randomized controlled trial (RCT) in children ages 6–12, the Micronutrients for ADHD in Youth (MADDY) study, indicated that children in the multinutrient group were three times more likely to have significantly improved behavioral symptoms compared to children in the placebo group [26]. To examine possible biological mechanisms of response, plasma samples from the MADDY RCT, collected at baseline and after 8 weeks of treatment with the multinutrients or placebo, were analysed for biomarkers of antioxidant status and oxidative stress (GPx and GR enzyme activities, BAP, and ROM, referred to hereafter as simply “OS biomarkers”) and trace mineral concentrations (including Cr, Cu, Fe, Mn, Se, and Zn). These factors have been assessed in separate analyses, finding that the multinutrients increased plasma Se and Cr (in females only) compared to placebo [27]. In addition, the multinutrients decreased ROM compared to placebo, and identified the change in ROM as a potential mediator of the behavioral improvements observed in the trial [28]. Given the critical role that trace minerals serve in antioxidant enzyme activity, the current analyses aimed to elucidate the relationship between trace minerals and OS measures in children with ADHD after 8-weeks of multinutrient supplementation by assessing both their contribution to antioxidant defense and free radical production.

The specific objectives were to: (1) determine if 8-week change in these trace minerals were correlated with the 8-week change in OS biomarkers, and (2) determine if baseline plasma mineral concentrations moderated the relationship between the treatment group (multinutrient or placebo) and the change in OS biomarkers during the 8-week RCT.

## Methods

The MADDY RCT primary outcomes of treatment response and safety profile along with details of study design have been published elsewhere [26, 29, 30]. Methods are described briefly below.

## Summary of MADDY RCT Design

The RCT was conducted at three sites (2 in the U.S. and 1 in Canada- although blood samples were only collected at the two U.S. sites) and children were randomized to either multinutrient or placebo group in a 3:2 ratio [26]. The multinutrient intervention was a commercially available blend of all known vitamins and minerals, plus amino acids and antioxidants, with nutrient dosages generally above the Recommended Dietary Allowance (RDA) and below Lowest Observed Adverse Effects Level (LOAEL) except as noted [31]. Table 1 provides a summary of the dosage of each of each mineral by age group, which includes all the minerals included in this analysis. The placebo contained 0.1 mg riboflavin per capsule to mimic urine color when supplemented with B-vitamins (for blinding) and cellulose filler to look identical to the multinutrient capsule. Further details about multinutrient formulation and inclusion/exclusion criteria were previously published [26], and are summarized in supplemental materials for reference including the supplement facts label with the full list of ingredients. Concomitant nutritional supplementation was permitted only if the supplement did not contain ingredients in the active intervention [26].

**Table 1:** Dosage of each mineral contained in multinutrient formulation and RDA/AI, UL, and LOAEL or NOAEL. Originally published in Robinette et al., 2024 [27] Abbreviations: LOAEL=Lowest Observed Adverse Effects Level; NOAEL=Lowest Observed Adverse Effects Level; RDA= Recommended Daily Allowance; AI= Adequate Intake, M=male, F=female, NS=not specified, ND=not determined, NE=not established.

Mineral			6–8-year-olds			9–12-year-olds			
	Unit	1 Cap	max dose (9 caps)	RDA or AI (4–8 years)	UL (4–8 years)	max dose (12 caps)	RDA or AI (9–13 years)	UL (9–13 years)	LOAEL
Chromium *(as NutraTek™ chelation complex)*	mcg	52	468	15 (AI)	ND	624	25 (M)/ 21 (F) (AI)	ND	NE
Copper *(as NutraTek™ chelation complex)*	mg	0.6	5.4	0.44	3	7.2	0.7	5	10 (NOAEL)
Iodine *(as NutraTek™ chelation complex)*	mcg	17	153	90	300	204	120	600	1700
Iron *(as NutraTek™ chelation complex)*	mg	1.15	10.35	10	40	13.8	8	40	70
Magnesium *(as NutraTek™ chelation complex)*	mg	50	450	130	110	600	240	350	360
Manganese *(as NutraTek™ chelation complex)*	mg	0.8	7.2	1.5 (AI)	3	9.6	1.9 (M)/ 1.6 (F) (AI)	6	15
Molybdenum *(as NutraTek™ chelation complex)*	mcg	12	108	22	600	144	34	1100	1500
Phosphorus *(as NutraTek™ chelation complex)*	mg	70	630	500	3000	840	1250	4000	10,200
Selenium *(as NutraTek™ chelation complex)*	mcg	17	153	30	150	204	40	280	913
Zinc *(as NutraTek™ chelation complex)*	mg	4	36	5	12	48	8	23	60
Boron *(as NutraTek™ chelation complex)*	NS								NE
Lithium *(as NutraTek™ chelation complex)*	NS								NE
Nickel *(as NutraTek™ chelation complex)*	NS								NE
Vanadium *(as NutraTek™ chelation complex)*	NS								NE

At the baseline visit, written informed consent was obtained from all parents/guardians and assent from children prior to any study procedures. The RCT was prospectively registered in the ClinicalTrials.gov database (NCT 03252522) and approved by the US Food and Drug Administration (FDA) under an investigational new drug application (IND #127832) and by Health Canada (Control #207742). All procedures involving human subjects were approved by Institutional Review Boards (IRB) at The Ohio State University (OSU) (IRB # 2017H0188), Oregon Health & Science University (OHSU) (IRB # 16870), and the Conjoint Health Research Ethics Board at University of Calgary (REB# 17–0325) for the University of Lethbridge. The trial was conducted according to the guidelines in the Declaration of Helsinki.

### Sample Size

**Fig. 1:**
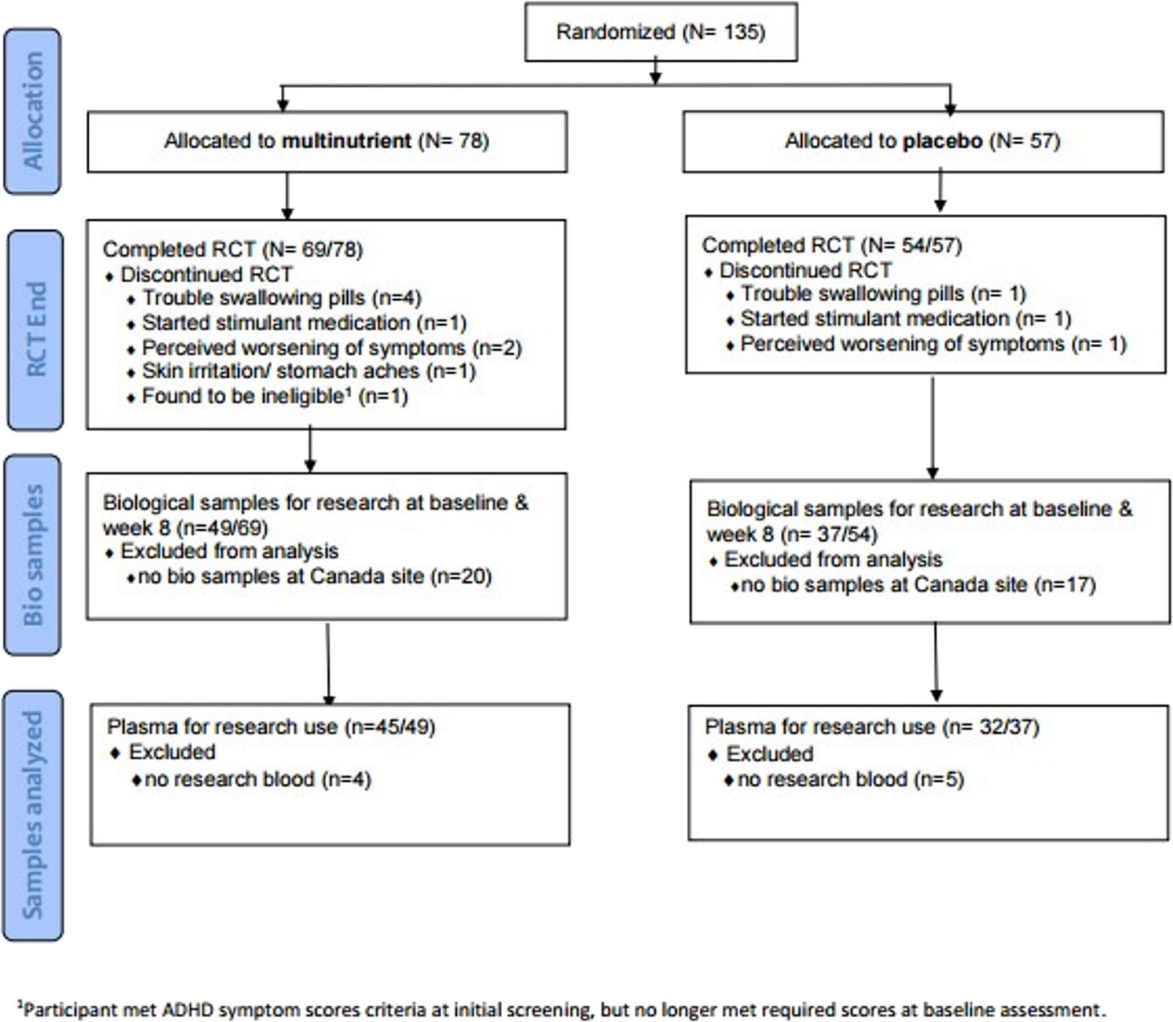
CONSORT flow diagram for MADDY RCT updated to include biological samples used in these analyses. Adapted from Johnstone et. al., 2022 [26]. Abbreviations: RCT: randomized controlled trials; ADHD: attention-deficit/hyperactivity disorder

Sample size was determined by the availability of frozen plasma samples from U.S. participants at baseline and end of the RCT (week 8) for this secondary analysis (Fig. 1). Frozen plasma samples from baseline and week 8 were analyzed for oxidative stress biomarkers and trace minerals concentrations for *n* = 71 participants (44 multinutrient, 27 placebo) [27, 28]. Missing samples were primarily due to insufficient quantity of blood drawn during the visit [27, 28].

### Measures

#### Demographic and Anthropometric Measures

Anthropometric measures of height and weight were measured at baseline and week 8 visits using a stadiometer with adjustable headpiece and a calibrated digital scale. Body mass index (BMI) was calculated based on height and weight. Demographic characteristics were assessed via parent questionnaire at the baseline visit.

#### Trace Mineral Concentrations

Detailed descriptions of plasma mineral concentration determination and results have been previously published [27]. Data generation methods are summarized here. Blood was collected, processed, and separated into plasma using standard methods and then aliquoted and stored at −80 °C for future mechanistic analyses [27]. Plasma trace minerals were measured using Inductively Coupled Plasma Mass Spectroscopy (ICP-MS) performed on an Agilent 7700x equipped with an ASX 500 autosampler at the Oregon Health & Science University (OHSU) Elemental Analysis Shared Resource [27]. An internal standard (scandium, germanium, bismuth) continuously introduced with the sample was used to correct for detector fluctuations and to monitor plasma stability [27].

#### Oxidative Stress

Detailed procedures and results are published [28]. An Agilent Biotek Synergy H1 Microplate Reader was used to measure absorbance on baseline and week 8 plasma samples [28]. Each assay is described briefly below.

*Biologic Antioxidant Potential (BAP)* was measured using Diacron BAP kit and is reported in units of µmol/L [32]. It is a measure of the total non-enzymatic antioxidant capacity of blood plasma [28]. Absorbance was measured at 505 nm.

*Reactive oxygen metabolites (ROM)* were measured using Diacron derivatives of ROM kit and is reported in Caratelli Units (UCARR), with 1 UCARR equivalent to 0.08 mg H_2_O_2_/dL [33]. It is a measure of the amount of hydroperoxides (a stable product of the lipid peroxidation process) in a sample using an iron-mediated reaction. Absorbance was measured after 90 min of incubation at 505 nm [28].

*Glutathione reductase (GR) enzyme activity* was measured using Cayman Chemicals Glutathione Reductase Assay Kit (703202) [34]. Activity was quantified as nmol/min/mL. This kit works by measuring the rate of nicotinamide adenine dinucleotide phosphate (NADPH) oxidation, which is accompanied by a decrease in absorbance at 340 nm and is directly proportional to the GR activity in the sample [28]. Absorbance was monitored at 340 nm once every minute to obtain at least 5 time points [28].

*Glutathione Peroxidase (GPx) enzyme activity* was measured using Cayman Chemicals Glutathione Reductase Assay Kit (703102) which measures GPx activity indirectly by a coupled reaction with GR, in which GSSG that is produced upon reduction of hydroperoxide by GPx is recycled back to its reduced state by GR and NADPH [35]. The oxidation of NADPH to NADP + is accompanied by a decrease in absorbance at 340 nm [28]. Absorbance was measured at 340 nm once every minute to obtain at least 5 timepoints [28]. Activity was quantified as nmol/min/mL.

### Statistical Methods

Participant characteristics were reported as mean and standard deviation (SD) for data with normal distribution, median and interquartile range (IQR) for data that were not normally distributed and frequency (percent) for categorical variables. Each variable was reported for the total analysis cohort and compared between intervention arms using Mann-Whitney U test for continuous non-parametric variables and the Pearson chi-squared or Fisher’s exact test (if expected counts < 5) for categorical variables.

Any measured value of OS and trace minerals that was below detection limits was coded as half (½) of the detection limit for data analysis, following published methods [36]. For each participant, change after the 8-week RCT was calculated for each OS biomarker using the value at week 8 minus the value at baseline (“8-week change”), and for each trace mineral using the value at week 8 minus the value at baseline divided by baseline value * 100 (“8-week percent change”).

For objective 1, Pearson’s (for parametric variables) or Spearman’s correlations (for non-parametric variables) were conducted between each OS biomarker 8-week change and each trace mineral 8-week % change for the entire sample and by treatment group.

For objective 2, ordinary least squares (OLS) linear regression models were used as the moderation models following the analytical framework described by Kraemer et al. (2002) for moderation analysis in an RCT [ [38]. Unadjusted regression models predicted the outcome (O, 8-week change in each OS biomarker) with independent variables of treatment group (T, multinutrient or placebo group), the potential moderator (M, baseline mineral concentration), and the multiplicative interaction variable (i.e., TxM) [Eq. 1: O = a*M + b*T + c(M*T)] [] [38]. The baseline mineral concentration was considered a moderator if the unstandardized regression coefficients (β) of the interaction term (represented by c in Eq. 1) reached significance (*p* < 0.05); or was considered an independent predictor when the regression coefficient of the mineral independent term (represented by coefficient a in Eq. 1) reached significance. Significant interactive effects were explored graphically to understand the direction of moderation. Each baseline mineral concentration was mean-centered prior to inclusion in the model to avoid multicollinearity. Treatment groups were coded as −0.5 for placebo and 0.5 for multinutrient as recommended by Kraemer et al. (2002) [38].

Se was evaluated as a potential moderator for GR and GPx due to the direct role of Se in glutathione. All relevant minerals (Cr, Cu, Fe, Mn, Se, and Zn) were evaluated as potential moderators of BAP and ROM due to potential occurrence of multiple enzymatic and non-specific reactions influencing these measures. Each relevant outcome and baseline mineral relationship was evaluated with separate regression equations. Adjusted regression models were conducted with additional covariates of study site, sex, and baseline value of the OS biomarker. Assumptions for linear regression were tested for each OLS regression model using graphical and statistical methods including Shapiro-Wilks test for normality of residuals, White’s test for homoscedasticity of residuals, and variance inflation factors (VIFs) to assess independent variables for multicollinearity. Cook’s D test measured influence of individual observations; to determine if significant findings were influenced by any highly influential observations, those observations were excluded and regressions re-run.

All analyses were conducted using Stata version 18 software (College Station, TX: StataCorp LLC) [37]. Statistical significance was defined as a two-sided p-value < 0.05 for these exploratory analyses.

## Results

### Study Population Characteristics

The participants included in these analyses (*n* = 71; 44 multinutrient, 27 placebo) were 70% male, and predominantly White and non-Hispanic, with a median age of 10.4 years (IQR: 8.9–11.2) and BMI of 16.7 kg/m^2^ (IQR: 15.5–19.2) (Table 2). These characteristics were not different between the two treatment groups, except for sex, in which the placebo group had a higher proportion of females compared to the multinutrient group (*p* = 0.007).

**Table 2:** Characteristics of the study population comparing multinutrient and placebo groups

Characteristics	Total (*n* = 71)	Multinutrient (*n* = 44)	Placebo (*n* = 27)	*p*-value^b^
Child’s Age (years), median (IQR)	10.4 (8.9–11.2)	10.5 (8.7–11.3)	10.4 (9.1–11.1)	0.542
BMI (kg/m2), median (IQR)	16.7 (15.5–19.2)	16.8 (15.7–18.9)	16.5 (14.7–19.5)	0.425
Child’s Sex, n (%)				0.007
Male	50 (70.4)	36 (81.8)	14 (51.8)	
Female	21 (29.6)	8 (18.2)	13 (48.2)	
Family Income, annually, n (%)				0.565
< $30,000	6 (8.5)	4 (9.1)	2 (7.4)	
$30,001–60,000	11 (15.5)	5 (11.4)	6 (22.2)	
$60,001–80,000	9 (12.7)	7 (15.9)	2 (7.4)	
≥ $80,001	45 (63.4)	28 (63.6)	17 (63.0)	
Parent Marital Status, n (%)				0.416
Married	52 (73.2)	31 (70.5)	21 (77.8)	
Divorced	15 (21.1)	9 (20.5)	6 (22.2)	
Single	4 (5.6)	4 (9.1)	0 (0)	
Parent Educational Level, n (%)				0.917
High school	5 (7.0)	3 (6.8)	2 (7.4)	
Technical college/trade school	15 (21.1)	10 (22.7)	5 (18.5)	
University or higher	51 (71.8)	31 (70.5)	20 (74.1)	
Ethnicity^a^, n (%)				0.160
Not Hispanic or Latino	48 (67.6)	27 (61.4)	21 (77.8)	
Hispanic or Latino	6 (8.5)	6 (13.6)	0 (0)	
Other^c^	2 (2.8)	1 (2.3)	1 (3.7)	
Race^a^, n (%)				0.692
Asian	2 (2.8)	2 (4.5)	0 (0)	
Black	6 (8.5)	3 (6.8)	3 (11.1)	
White	60 (84.5)	36 (81.8)	24 (88.9)	
Other ^d^	1 (1.4)	1 (2.3)	0 (0)	

Abbreviations: IQR: interquartile range; SD: standard deviation; BMI: Body Mass Index

^a^ 15 participants did not report ethnicity and 2 participants did not report race

^b^ P-values comparing multinutrient to placebo groups using Mann-Whitney U-test for non-parametric continuous variables and the Pearson chi squared or Fisher’s exact test (if expected counts < 5) for categorical variables

^c^ Includes Jewish, Japanese, or other for ethnicity

^d^ Includes American Indian/Native American or Alaska Native, Métis, Native Hawaiian/Pacific Islander or other for Race

### Correlations Between Baseline Plasma Trace Minerals and OS biomarkers

Correlations between baseline plasma trace minerals and baseline OS biomarkers are reported in Table 3. Baseline plasma Cu was correlated with baseline ROM (r = 0.54, p<0.001). Therefore, baseline Cu was included as covariate in ROM moderation models. 

**Table 3:** Correlation coefficients for each baseline plasma trace mineral concentration with each baseline plasma OS biomarker

Baseline trace mineral concentrations	GPx	GR	BAP	ROM
	*r* (*p*-value)	*r* (*p*-value)	*r* (*p*-value)	*r* (*p*-value)
Plasma (µg/L)				
Chromium	−0.11 (0.36)	0.03 (0.78)	0.01 (0.93)	−0.05 (0.67)
Copper	−0.15 (0.23)	0.00 (0.99)^a^	−0.09 (0.44)^a^	**0.54 (0.00)**
Iron	0.21 (0.07)	0.07 (0.56)	−0.01 (0.93)	−0.03 (0.80)
Manganese	0.11 (0.37)	0.04 (0.71)	−0.12 (0.34)	0.02 (0.86)
Selenium	−0.02 (0.88)	−0.05 (0.66)^a^	0.05 (0.69) ^a^	0.07 (0.55)
Zinc	−0.10 (0.40)	0.06 (0.64)	0.10 (0.42)	0.08 (0.53)

Abbreviations: r: correlation coeffient; BAP: biological antioxidant potential; ROM: reactive oxygen metabolites; OSI: oxidative stress index; GR: glutathione reductase; GPx: glutathione peroxidase; UCarr: Carratelli units; ug/L: microgram per liter.

^a^ Pearson’s correlation coefficient, all others Spearman correlation coefficients.

### Correlations Between Mineral 8-Week % Changes and OS 8-Week Changes

GPx 8-week change was positively correlated with Se 8-week % change overall (r=0.26, p=0.03), with similar strength of correlation in both the multinutrient and placebo groups, though neither group reached significance when considered separately (Table 4). GR 8-week change was positively correlated with Cu 8-week % change in multinutrient group (r=0.37, p=0.01), but negatively correlated in placebo group (r=−0.49, p=0.009); the correlation was not significant in the total sample. BAP 8-week change in the multinutrient group only was positively correlated with Zn 8-week % change (r=0.37, p=0.01) and negatively correlated with Cr 8-week % change (r=−0.41, p=0.005), with no significant correlations identified within the placebo group or the total sample. ROM 8-week change was positively correlated with Cu 8-week % change (r=0.51, p<0.001) and negatively correlated with Mn 8-week % change (r=−0.24, p=0.05) in the total sample. Within the multinutrient group, ROM 8-week change was positively correlated with Cu and Se 8-week % change (r=0.58, p<0.001 and r=0.34, p=0.03, respectively) and negatively correlated with Mn 8-week % change (r=−0.30, p=0.04). No correlations with ROM were significant in the placebo group. 

**Table 4:** Correlation coefficients between each plasma trace mineral 8-week percent change with each OS biomarker 8-week change, for the sample overall and by treatment group

	GPx 8-week change			GR 8-week change			BAP 8-week change			ROM 8-week change		
	overall	multi-nutrient	placebo	overall	multi-nutrient	placebo	overall	multi-nutrient	placebo	overall	multi-nutrient	placebo
Plasma trace mineral 8-week percent change	r (p-value)	r (p-value)	r (p-value)	r (p-value)	r (p-value)	r (p-value)	r (p-value)	r (p-value)	r (p-value)	r (p-value)	r (p-value)	r (p-value)
chromium	0.21 (0.07)	0.28 (0.07)	0.07 (0.71)	0.13 (0.29)	0.08 (0.61)	0.28 (0.16)	−0.23 (0.06)	**−0.41 (0.005)**	0.04 (0.85)	−0.15 (0.21)	0.03 (0.84)	−0.31 (0.11)
copper	0.15 (0.22)	0.15 (0.33)	0.19 (0.35)	0.09 (0.44)	**0.37 (0.01)**	**−0.49 (0.009)**	−0.21 (0.08)	−0.18 (0.25)	−0.30 (0.13)	**0.51 (0.000)**	**0.58 (0.000)**	0.33 (0.09)
iron	0.07 (0.56)	0.14 (0.38)	0.00 (1.00)	0.02 (0.90)	−0.06 (0.72)	0.13 (0.53)	0.18 (0.13)	0.05 (0.76)	0.37 (0.06)	0.00 (0.98)	−0.26 (0.09)	0.26 (0.19)
manganese	−0.17 (0.17)	−0.12 (0.44)	−0.21 (0.30)	−0.03 (0.79)	−0.24 (0.12)	0.35 (0.08)	−0.08 (0.51)	−0.11 (0.46)	−0.01 (0.98)	**−0.24 (0.05)**	**−0.30 (0.04)**	−0.21 (0.30)
selenium	**0.26**^**a**^ **(0.03)**	0.24 ^a^ (0.12)	0.35 ^a^ (0.07)	0.00 (1.00)	0.21 (0.18)	−0.23 (0.26)	0.11 ^a^ (0.38)	0.16 ^a^ (0.29)	0.04 ^a^ (0.85)	0.18 ^a^ (0.14)	**0.34** ^**a**^ **(0.03)**	0.25 ^a^ (0.21)
zinc	0.05 ^a^ (0.69)	−0.07 ^a^ (0.65)	0.24 ^a^ (0.23)	−0.11 (0.37)	−0.05 (0.74)	−0.15 (0.46)	0.04 ^a^ (0.77)	**0.37** ^**a**^ **(0.01)**	−0.32 ^a^ (0.11)	−0.16 ^a^ (0.17)	−0.14 ^a^ (0.35)	−0.06 ^a^ (0.76)

Abbreviations: BAP: biological antioxidant potential; ROM: reactive oxygen metabolites; OSI: oxidative stress index; GR: glutathione reductase; GPx: glutathione peroxidase

^a^ Pearson’s correlation coefficient, all others Spearman correlation coefficients

### Baseline Trace Mineral Concentrations as Moderators of Oxidative Stress 8-Week Change

**Fig. 2:**
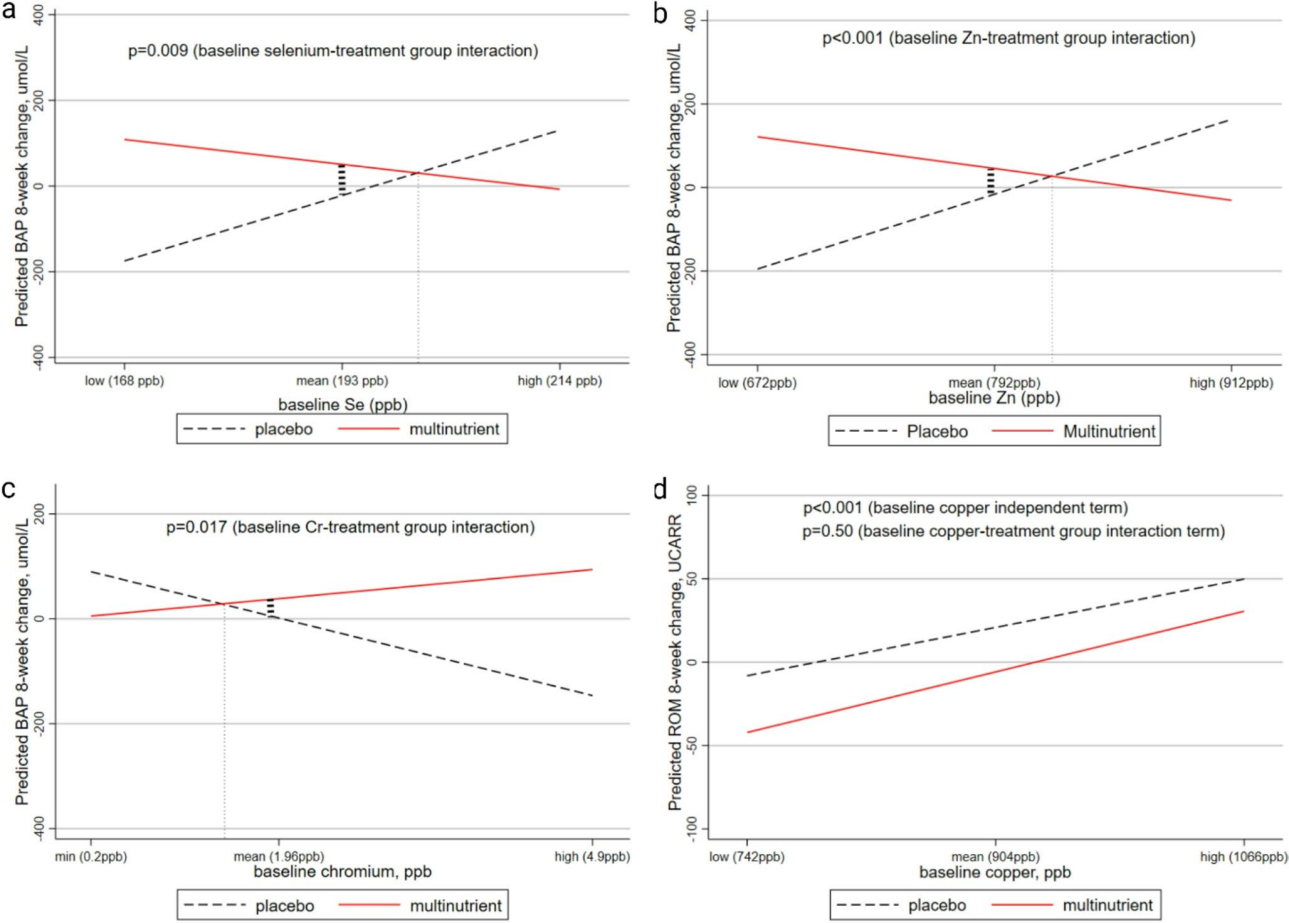
Baseline mineral concentration as moderators or independent predictors of treatment on BAP or ROM 8-week change. Trend lines represent regression of BAP 8-week change on baseline mineral concentrations (Se, Zn, Cr,) or ROM 8-week change on Cu, for each treatment group. The vertical separation between the two lines at each value of baseline mineral concentration indicates the size of the effect of treatment on BAP or ROM 8-week change for that subpopulation. The main effect of treatment on BAP 8-week change is depicted by the dashed vertical line above the mean baseline concentration: a. Se (193 ppb), b. Zn (792 ppb), c. Cr (2.0 ppb). The location where the two trend lines cross indicates the mineral’s baseline value for Se (~200 ppb) and Zn (~820 ppb) below which the multinutrient group is more effective at increasing BAP than placebo. For Cr, the location where the two trend lines cross indicates the baseline Cr (~1.5 ppb) above which the multinutrient group is more effective at increasing BAP than placebo. The main effect of treatment on ROM 8-week change is depicted by the dashed vertical line above the mean baseline concentration: d. Cu (904 ppb). Abbreviations: BAP: biological antioxidant potential, ROM: reactive oxygen metabolites, ppb: parts per billion, µmol/L: micromole/liter, UCARR: Caratelli units.

Linear regression coefficients for the moderation models for each OS biomarker 8-week change are shown in Table 5. In both the unadjusted and adjusted BAP models, baseline Se, Zn, and Cr were moderators of the effect of treatment group on BAP 8-week change: baseline Se-by-treatment group interaction (adjusted: β=−9.45, p=0.006), baseline Zn-by-treatment group interaction (adjusted: β=−2.13, p<0.001), and baseline Cr-by-treatment group interaction (adjusted: β=68.9, p=0.017) [38]. Notably, the direction of the Cr response was distinct from Zn and Se. Figure 2 illustrates the direction of the moderation effects. In the multinutrient group, children with low Se, low Zn, and high Cr at baseline were likely to have an increase in BAP after 8-weeks compared to those with high baseline Se, Zn, and low Cr; the trend was opposite in the placebo group (Figure 2a, b, c). In the figures, the location of the intersection of the treatment group lines indicates the levels of baseline Se, Zn, and Cr at which the multinutrient is more effective than placebo at increasing BAP. This occurs below baseline Se of 200 ppb, below baseline Zn of 820 ppb, and above baseline Cr at 1.5 ppb; indicating subgroups of children defined by their baseline plasma mineral concentrations that benefitted from multinutrients with increased antioxidant potential.

**Table 5:** Baseline trace minerals as potential moderators or independent predictors of OS biomarker 8-week changes

		Model 1 (unadjusted)				Model 2 (adjusted)			
Outcome	Potential Moderator	mineral independent term	mineral x treatment group interaction	model fit		mineral independent term	mineral x treatment group interaction	model fit	
8-week change in OS	mineral baseline concentration	beta (p-val)	beta (p-val)	F (df)	Prob > F	beta (p-val)	beta (p-val)	F (df)	Prob > F
**GPx**	selenium	0.0381 (0.68)	0.136 (0.46)	0.36 (3, 67)	0.78	0.0572 (0.60)	0.0666 (0.72)	1.52 (6, 64)	0.18
**GR**	selenium	0.0153 (0.72)	−0.0942 (0.27)	0.92 (3, 67)	0.43	−0.000365 (0.99)	−0.0760 (0.26)	8.50 (6, 64)	< 0.001
**BAP**	chromium	−11.0 (0.43)	**64.0 (0.025)**	1.82 (3, 67)	0.15	−15.6 (0.30)	**68.9 (0.017)**	1.54 (6, 64)	0.18
	copper	0.078 (0.75)	−0.0057 (0.99)	0.04 (3, 67)	0.99	0.082 (0.76)	−0.115 (0.82)	0.50 (6, 64)	0.81
	iron	0.075 (0.38)	−0.20 (0.23)	0.54 (3, 67)	0.66	0.12 (0.20)	−0.21 (0.23)	0.87 (6, 64)	0.53
	manganese	79.2 (0.60)	−160 (0.60)	0.10 (3, 67)	0.96	136 (0.40)	−260 (0.42)	0.60 (6, 64)	0.73
	selenium	2.52 (0.13)	**−8.26 (0.014)**	2.33 (3, 67)	0.082	2.58 (0.19)	**−9.45 (0.006)**	1.95 (6, 64)	0.09
	zinc	0.41 (0.15)	**−2.05 (0.001)**	5.29 (3, 67)	0.003	0.43 (0.17)	**−2.13 (< 0.001)**	3.35 (6, 64)	0.006
**ROM**	chromium	5.66 (0.06)	−11.4 (0.06)	4.99 (3, 67)	0.003	0.35 (0.87)	−4.8 (0.23)	17.09 (8, 62)	< 0.001
	copper	−0.075 (0.14)	−0.069 (0.50)	4.46 (3, 67)	0.006	**0.202 (< 0.001)**	0.046 (0.50)	19.28 (7, 63)	< 0.001
	iron	−0.0077 (0.67)	0.026 (0.46)	3.39 (3, 67)	0.02	−0.015 (0.22)	0.021 (0.36)	17.09 (8, 62)	< 0.001
	manganese ^a^	3.92 (0.90)	−10.0 (0.88)	3.20 (3, 67)	0.03	28.3 (0.29)	−21.9 (0.64)	16.96 (8, 61)	< 0.001
	selenium	−0.108 (0.77)	−0.414 (0.57)	3.40 (3, 67)	0.03	0.0121 (0.66)	0.423 (0.38)	16.89 (8, 62)	< 0.001
	zinc	−0.0239 (0.72)	−0.066 (0.62)	3.31 (3, 67)	0.03	0.0453 (0.34)	0.00204 (0.98)	16.78 (8, 62)	< 0.001

Model 1: includes treatment group, baseline mineral, and treatment group x baseline mineral interaction terms

Model 2: Model 1 plus site, sex, and baseline value of the outcome variable (plus Cu 8-week % change and baseline Cu for ROM models only)

^a^ model excludes one participant to meet linear regression assumptions

No minerals were moderators or independent predictors of ROM 8-week change in the unadjusted models. In the adjusted models, baseline Cu was an independent predictor of ROM 8-week change (β=0.202, p<0.001): baseline Cu was positively associated with ROM 8-week change in both treatment groups (Figure 2d). Fe and Mn were not moderators or independent predictors of 8-week change in ROM or BAP. Baseline Se was not a moderator of 8-week change in GPx or GR.

## Discussion

These analyses examine the relationships between plasma trace mineral concentrations and OS changes in children with ADHD and emotional dysregulation, following 8 weeks of multinutrient or placebo supplementation. A major finding is that plasma Se concentrations contributed to both antioxidant activity, as hypothesized, and oxidative stress, which was unexpected. The contribution of Se to antioxidant activity is evidenced by significant trends identified with BAP and GPx. First, the 8-week change in GPx enzyme activity was positively correlated with Se 8-week percent change in both treatment groups. This suggests that plasma GPx activity primarily depends on the available plasma Se levels in all participants, independent of whether they received Se supplementation or not. Second, baseline plasma concentrations of Se moderated the relationship between the treatment group and 8-week change in BAP. An important purpose of moderation analysis in an RCT is to reveal efficacy of a treatment among different subgroups [38]. In the multinutrient group, children with low baseline Se experienced an increase in BAP after 8-weeks of supplementation, indicating an improved ability to protect against ROS, while those with high baseline Se showed no change in BAP. This contrasts with the placebo group, in which children with low baseline Se experienced a decrease in BAP, while those with high baseline Se experienced an increase in BAP after 8-weeks. Children with baseline plasma Se levels below about 200ppb benefitted with a larger increase in BAP after 8-weeks of multinutrient compared to placebo, while children with baseline Se above 200ppb did not exhibit antioxidant benefit from the multinutrient. The potential mechanism for the antioxidant benefit could include Se’s key role in the enzymes recycling glutathione, a major endogenous antioxidant, aligning with the observation that participants with adequate physiological levels of Se did not further benefit from supplementation. Meanwhile, the presence of riboflavin in the placebo (~ 1 mg if taking 9–12 capsules daily) is hypothesized to have increased the antioxidant ability of glutathione [39], influencing the BAP response in placebo group participants with sufficient levels of Se.

Importantly, however, plasma Se was also related to oxidative stress: ROM 8-week change was positively correlated with Se 8-week change within the group of children taking the multinutrient, but not placebo. This finding is counter-intuitive given that an increase in Se was related to an increase in GPx activity, which presumably would reduce production of ROS. This finding potentially supports previous research that indicates a U-shaped curve for the health effects of supplemental Se, where supplemental Se may benefit people with low status, while those with adequate-to-high status might be adversely affected and should avoid Se supplementation [40]. Se supplementation has been tested as treatment for many diseases in adulthood, including joint diseases [41, 42], thyroid disease [43], diabetes and cancers [40], etc., although few of these studies evaluate for U-shaped dose-response relationships. Rayman (2012) suggests that a baseline plasma Se value of 122 ug/L delineates a change in risk of cancer and type 2 diabetes when supplementation with 200 mg of Se per day is added, thus increasing risk [40]. One meta-analysis of Se supplementation effect on immune function found an inverted U-shape relationship with NK cell count [44]. Another found a non-linear relationship between Se dose and fasting blood sugar, where Se decreased fasting blood sugar until the trend reversed at doses above 200 mg/day [45]. Although our findings from the correlation analysis are exploratory and do not necessarily indicate adverse health effects, these results suggest that children may also have a U-shaped curve of health benefit from Se supplementation based on their current Se levels, although the level at which this occurs may be different from adults and should be further studied and defined.

Our findings revealed additional relationships between Zn and Cr with antioxidant activity. First, BAP 8-week change was positively correlated with Zn 8-week percent change and negatively correlated with Cr 8-week percent change amongst the multinutrient group only. Second, baseline plasma Zn and Cr moderated the 8-week change in BAP, such that children with low baseline plasma Zn (below ~ 820 ppb) and/or high baseline plasma Cr concentrations (above ~ 1.5 ppb) benefited with an increase in BAP after 8-weeks of multinutrient compared to placebo. In our previously published analysis, we found that Zn increased after 8-weeks in the multinutrient group [27]. A potential mechanism through which increased Zn may increase antioxidant activity is through its interactions with metallothionein, a strong hydroxyl radical scavenger [46]. Zn^2+^ activates metal responsive transcription factor-1 (MTF-1) and upregulates metallothionein synthesis, plus Zn^2+^ binding to metallothionein protects metallothionein from degradation; therefore, supplementation with Zn may increase metallothionein synthesis and subsequently antioxidant capacity [46]. Secondly, Zn is an important structural component of Cu/Zn-SOD, and increased Zn concentrations may improve this enzyme’s antioxidant capacity [13, 47, 48], although antioxidant enzyme activities are not directly assessed by the BAP assay. Finally, the negative correlation between Cr and BAP 8-week changes could potentially be related to transferrin, an iron-binding transport protein that exhibits antioxidant capability by readily binding with free Fe^3+^ which reduces their concentrations and utility in Fenton reactions [49]. Cr competes with Fe for transferrin binding sites; therefore, lower concentrations of Cr could theoretically enable higher capacity for transferrin to bind free Fe [50]. Further, Cr impairs SOD activity, further reducing antioxidant protections [14]. Regardless of the mechanism, the negative correlation between Cr and BAP suggests Cr contributes to OS by reducing antioxidant capacity.

Finally, ROM 8-week change was negatively correlated with Mn 8-week % change in the multinutrient group only, which theoretically could be related to increased activity of Mn-SOD antioxidant enzyme, which neutralizes the more damaging superoxide radical into hydrogen peroxide specifically in the mitochondria [51]. It is likely that Mn-SOD enzyme activity is not assessed by the BAP assay measuring total antioxidants in plasma; however, its inverse association with ROM is probably due to reducing mitochondrial radical load.

These analyses also revealed that plasma Cu plays a crucial role in OS as measured by ROM. In addition to baseline plasma Cu being an independent predictor of ROM 8-week change, this study found that baseline plasma Cu was positively correlated with baseline ROM, and Cu 8-week % change was positively correlated with ROM 8-week change. These results suggest that Cu is an important confounder of ROM measurement. This direct relationship between Cu and ROM may be partially due to the measurement technique for ROM based on the Fenton reaction. Ceruloplasmin levels, the copper-containing protein in the blood, appear to not be affected in ADHD patients [52]; nonetheless it has been debated as a potential source of interference in the ROM test [53, 54] accounting for a modest 20% of ROM variation in linear regression models [54]. In our study, the concentration of Cu in plasma explained about 50% of the variability in ROM according to both the baseline and 8-week change correlation coefficients. These results indicate the importance of plasma concentrations of Cu when measuring ROM and OS in children. Future studies could employ ROM to provide a sensitive method for detection of Cu impact on oxidative stress.

Together, these findings suggest several research directions to investigate proposed hypotheses. Future multinutrient studies of children with ADHD could stratify by baseline Se or Zn status to formally test the hypothesis that those with low Se/Zn benefit from the multinutrient through increased antioxidant potential, and measure SOD enzyme activity and metallothionein to verify whether concentration increases after treatment. Additionally, the change in plasma minerals and other hypothesized mediator variables should be measured at a midpoint of the trial to allow accurate analysis of cause-and-effect relationships. The major implication of this study is the demonstration of concentration-dependent effects of trace elements, which can shift from beneficial at moderate levels to adverse at higher concentrations, as is notably observed with selenium supplementation. A concentration-dependent relationship was also observed with other trace elements affecting various aspects of oxidative stress: chromium suppressed antioxidant potential, while manganese reduced pro-oxidant effects. Larger trials adjusting basal trace element levels in individuals with ADHD through personalized supplementation could be proposed for future research. Finally, these findings may prompt further multidisciplinary in vitro or in vivo studies to validate the proposed hypothetical biological mechanisms.

### Strengths and Limitations

A primary strength of this analysis is the use of the placebo group comparator to examine moderation effects in the context of an RCT, which minimized risk of bias due to randomization and blinding in the trial. A second strength is the inclusion of both antioxidant and oxidant measures to understand the effects of trace minerals on both sides of the oxidative stress balance.

There are several limitations. Since changes in minerals and OS biomarkers were measured at the same time points, the mineral 8-week changes could not be evaluated as mediators of the OS changes. Therefore, we examined correlations between these variables, which cannot prove causation. Future studies could measure changes in plasma mineral concentration at mid-point in the RCT to be evaluated as potential mediators. Peripheral measures of trace minerals and OS may not reflect what is occurring in the central nervous system that may affect ADHD symptoms. Since moderation analyses and correlations were conducted to generate hypotheses, we did not control for multiple testing, which increases the risk of Type I errors. Because this study is a *post hoc* secondary analysis and likely not sufficiently powered to detect interaction effects, it may not have detected all moderation relationships (Type II error). Additionally, since there are other antioxidants (vitamin C, vitamin E, alpha lipoic acid, etc.) in the multinutrient formulation that were not measured in the plasma, it is possible that they contributed to the changes in OS. Furthermore, the minerals in this formulation are part of a proprietary chelation complex and the exact chemical form or ligand for each mineral is not specified. Since some organic chelates such as picolinate (commonly used as a ligand for Cr in supplements) have evidence of causing DNA damage in cell cultures [55], the ligands themselves cannot be ruled out as being a contributor to oxidative stress. Finally, while this study found significant relationships between minerals and OS in this sample of children with ADHD, the generalizability of these results to other age groups or populations may be limited due to the specific characteristics of the study sample. These findings should be reproduced in other cohorts to confirm generalizability across other populations living with ADHD.

## Conclusions

Plasma trace mineral concentrations, especially Se, Cu, Cr, Mn, and Zn, impacted the oxidative stress balance in children with symptoms of ADHD taking multinutrients supplementation. Unlike a single nutrient supplement, the multinutrient supplement may exert benefit due to the synergistic action of a combination of nutrients that contribute to improved antioxidant activity in children with low baseline Se and Zn, or high baseline Cr, reducing the production of ROS from physiologically required trace minerals such as Cu. Further investigation is warranted to understand the effect of supplemental Se on children with adequate Se status.

## Supplementary Information

Below is the link to the electronic supplementary material.

Supplementary Material 1 (DOCX 180 KB)

## Data Availability

Data described in this manuscript will be made available upon reasonable request pending study team review.

## Abbreviation

ADHD: Attention-Deficit/Hyperactivity Disorder
BAP: Biological antioxidant potential
CASI-5: Child and Adolescent Symptom Inventory-5
CAT: Catalase
CGI-I: Clinical Global Impressions-Improvement scale
GPx: Glutathione peroxidase
GR: Glutathione reductase
GSH: Reduced glutathione
GSSG: Oxidized glutathione
MADDY: Micronutrients for ADHD in Youth Study
OS: Oxidative stress
RCT: Randomized controlled trial
ROM: Reactive oxygen metabolites
ROS: Reactive oxygen species
SOD: Superoxide dismutase
UCARR: Carratelli units
