# Supplementary material for: Association Between Trace Mineral Concentrations and Oxidative Stress in Children with ADHD Supplemented with Multinutrients
Source: Biol Trace Elem Res. 2026 Mar 3;204(7):5081–93. doi: 10.1007/s12011-026-05017-5 (PMC13320042; doi:10.1007/s12011-026-05017-5)
Supplement: Supplementary file 1 — Supplementary Material 1 (DOCX 180 KB) [file 12011_2026_5017_MOESM1_ESM.docx]

**SUPPLEMENTAL MATERIALS FOR**

“Association between trace mineral concentrations and oxidative stress in children with ADHD supplemented with multinutrients”

**Methodological Details of Micronutrients for ADHD in Youth (MADDY) RCT**

Details of MADDY RCT are described in detail in the primary outcomes manuscript (1) but summarized here to understand context for this secondary analysis:

Plasma mineral concentrations and oxidative stress data was generated from biological samples collected in the MADDY Study, an 8-week double blind RCT that examined the efficacy of a multi-vitamin/mineral supplement (referred to hereafter as multinutrient) as treatment for ADHD and emotional dysregulation in children aged 6-12. The primary outcomes of behavioural improvements and safety profile, along with details of study design are published elsewhere (1-3). Briefly, 6- to 12-year-old participants were recruited from three sites: two in the U.S. (Columbus, OH and Portland, OR) and one in Canada (Lethbridge, Alberta). The inclusion criteria were: 1) having 6 or more inattention and/or 6 or more hyperactivity/impulsivity symptoms on the parent-reported Child and Adolescent Symptom Inventory-5 (CASI-5) and at least one symptom of irritability or anger from the CASI-5 Oppositional Defiant Disorder or Disruptive Mood Dysregulation Disorder subscales, 2) being psychotropic-medication-free or washed-out for at least two weeks prior to the study, and 3) willingness to swallow 9-12 capsules per day and perform blood draws at baseline and end of RCT (4, 5). Exclusion criteria included any neurological disorder (e.g. intellectual disability, autism spectrum disorder) or other major psychiatric conditions requiring hospitalization (e.g. significant mood disorder, active suicidal ideation); any serious medical condition such as diabetes, hyperthyroidism, inflammatory bowel disease; and any known abnormality of mineral metabolism (e.g. Wilson disease, hemochromatosis) (1).

At the baseline visit, written informed consent was obtained from all parents/guardians and assent from children prior to any study procedures. The RCT was prospectively registered in the ClinicalTrials.gov database (NCT 03252522) and approved by the US Food and Drug Administration (FDA) under an investigational new drug application (IND #127832) and by Health Canada (Control #207742). All procedures involving human subjects were approved by Institutional Review Boards (IRB) at The Ohio State University (OSU) (IRB # 2017H0188), Oregon Health & Science University (OHSU) (IRB # 16870), and the Conjoint Health Research Ethics Board at University of Calgary (REB# 17-0325) for the University of Lethbridge. The trial was conducted according to the guidelines in the Declaration of Helsinki.

**Sample size**

For the RCT, 135 participants were recruited and 123 completed the RCT, with 123 being the sample size needed to detect differences between groups using a 3:2 randomization ratio of multinutrient to placebo (1).

**Intervention**

The intervention (multinutrient) consisted of a commercially available blend of all known vitamins and essential minerals, plus amino acids and antioxidants (product label available at manufacturer’s website: www.hardynutritionals.com/products/daily-essential-nutrients-360). A dose of 9 to 12 capsules per day provided nutrient dosages generally above the Recommended Dietary Allowance (RDA) and below Tolerable Upper Intake Levels (UL), though seven nutrients were above the UL, but below Lowest Observed Adverse Effect Level (LOAEL) [copper, folate, manganese, pyridoxine (B6), selenium, vitamin A (retinyl palmitate), zinc], and two above the LOAEL [magnesium and niacin (B_3_)] (6). Discussion of dosage and rationale for safety in this trial has been published (6). The placebo capsule looked identical to the multinutrient capsule and contained cellulose filler and 0.1mg of riboflavin per capsule (total dose 0.9-1.2mg/day, above RDA for this age group) added to mimic urine color when supplemented with B vitamins (1). Hardy Nutritionals (Raymond, AB, Canada) provided the intervention capsules, Daily Essential Nutrients, and the placebo capsules without cost, but had no role in the study design, data collection, analysis, or interpretation of the results.

**Figure S1: Supplemental Facts label from the manufacturer of the multinutrient supplement**


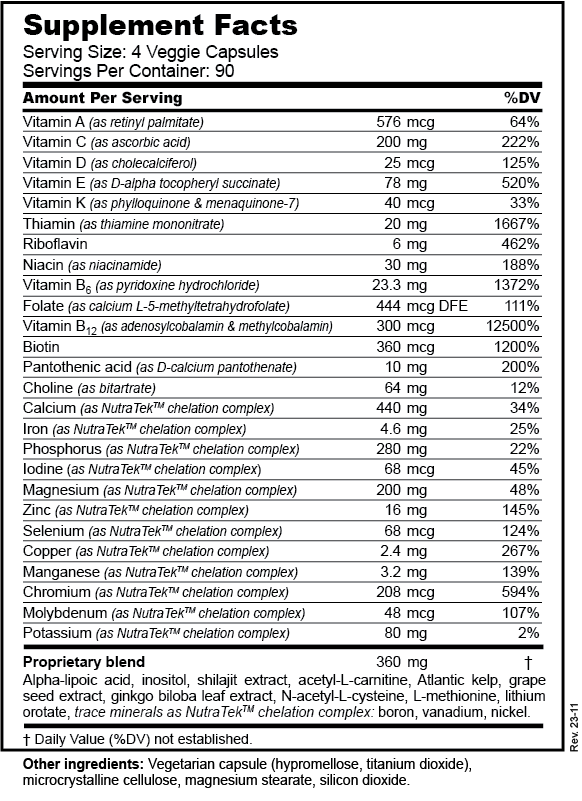


**References**

1. Johnstone JM, Hatsu I, Tost G, Srikanth P, Eiterman LP, Bruton AM, et al. Micronutrients for Attention-Deficit/Hyperactivity Disorder in Youths: A Placebo-Controlled Randomized Clinical Trial. J Am Acad Child Adolesc Psychiatry. 2022;61(5):647-61. doi: 10.1016/j.jaac.2021.07.005.

2. Johnstone JM, Leung B, Gracious B, Perez L, Tost G, Savoy A, et al. Rationale and design of an international randomized placebo-controlled trial of a 36-ingredient micronutrient supplement for children with ADHD and irritable mood: The Micronutrients for ADHD in Youth (MADDY) study. Contemporary clinical trials communications. 2019;16:100478.

3. Leung BMY, Srikanth P, Robinette L, Bruton AM, Tost G, Hatsu I, et al. Micronutrients for ADHD in youth (MADDY) study: comparison of results from RCT and open label extension. Eur Child Adolesc Psychiatry. 2023. doi: 10.1007/s00787-023-02236-2.

4. American Psychiatric Association. Diagnostic and Statistical Manual of Mental Disorders. 5th ed. Washington, DC, 2013.

5. Gadow KD, Sprafkin J. *Child and Adolescent Symptoms Inventory-5* [Internet]. Stony Brook, NY, Checkmate Plus; 2015; Available from: <https://www.checkmateplus.com/product/casi5.htm>.

6. Johnstone JM, Arnold LE, Villagomez A, Robinette LM, Gracious BL, Ast HK, et al. Dr. Johnstone et al. Reply to Dr. Elmrayed. Journal of the American Academy of Child & Adolescent Psychiatry. 2023. doi: <https://doi.org/10.1016/j.jaac.2023.07.994>.  .
